# Impact of implementing emergency demand response program and tie-line on cyber-physical distribution network resiliency

**DOI:** 10.1038/s41598-023-30746-1

**Published:** 2023-03-04

**Authors:** Sally R. Osman, Bishoy E. Sedhom, Sahar S. Kaddah

**Affiliations:** Electrical Engineering Department, Faculty of Engineering, Mansoura University, Mansoura, 35516 Egypt

**Keywords:** Electrical and electronic engineering, Energy grids and networks

## Abstract

Recently, due to the complex nature of cyber-physical distribution networks (DNs) and the severity of power outages caused by natural disasters, microgrid (MG) formation, distributed renewable energy resources (DRERs), and demand response programs (DRP) have been employed to enhance the resiliency of these networks. This paper proposes a novel multi-objective MGs formation method-based darts game theory optimization algorithm. The microgrid formation is obtained by controlling the sectionalizing and tie-line switches. The network graph theory is used to represent the constructed microgrid, and the non-linear equations of power flow and loss calculations are adopted in the microgrid formation model. To measure the system's resiliency under extreme disaster events, metrics are utilized to prove the system's flexibility and resiliency. The modified IEEE 33-bus test system is designed to validate the proposed approach's effectiveness. Three case studies are performed with and without considering the emergency demand response program (EDRP) and tie-lines.

## Introduction

**Figure 1:**
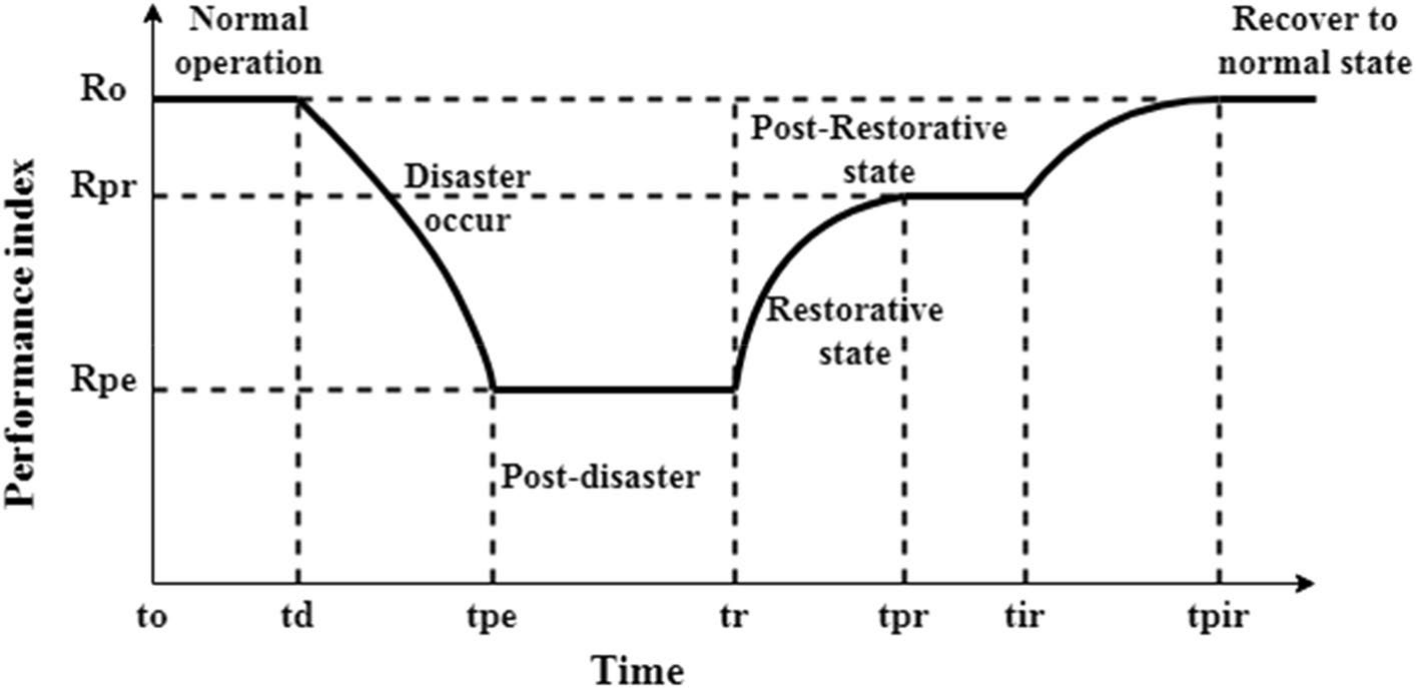
Conceptual resilience curve with a physical natural disaster.

With the increasing number of extreme weather catastrophes such as hurricanes, earthquakes, and floods, power utilities face many challenging issues, such as equipment failure, increased maintenance and operation costs, and inefficient operations, which may lead to system blackouts. While the growing advancement of distribution network information and communication technology, and the integration of distributed renewable energy resources (DRERs), smart networks can be described as complex cyber-physical distribution networks (DNs). The cyber-physical DNs include two networks: firstly, the physical network for power flow, and secondly, the cyber network for information flow. Recently, most blackouts have been caused by physical natural disasters, known as high-impact low, probability events^1^. These kinds of incidents cause massive losses and severe damage. For example, in the United States, the damage cost due to extreme weather events is about 25$-75$ billion a year^2^. Seven of the ten significant storms occurred in the last ten years, and each event caused damages of over 1-billion dollars^3^. Also, cyber-physical DNs are the most vulnerable to such events. Therefore, the importance of introducing a critical concept called "Resiliency" has been highlighted to complement other power system topics such as reliability, security, risk, and stability. The smart grid cyber-physical distribution network resiliency is the ability of the distribution network to resist, adapt, and rapidly recover in the presence of system disturbances^4^. Figure 1. demonstrates the changes in the performance index of a resilient network when facing a destructive event. In power systems, the reliability concept may confuse with resilience, but there are differences between the reliability and resilience concepts. The reliability is based on the operating point when the power system is static. In contrast, resilience is based on the network topology changes influenced by weather-related events. However, existing power systems are known to be reliable but not resilient^5^. Therefore, enhancing the network resiliency against natural disasters is considered the research gap in much energy sector research^6,7^. Breaking the distribution networks into self-supplied microgrids with connected distributed renewable energy resources to restore loads, especially critical ones, is a well-known procedure during major outages for improving cyber-physical resiliency through sectionalizing switches^11,17^.

Many researchers have introduced the microgrid formation strategy to enhance distribution network resiliency in inevitable natural disasters and achieve many objectives, such as loss minimization, operating cost reduction, load-shedding decrement, bus voltage regulation, and reliability improvement^18^. In^8–15^, the aftermath of natural disaster microgrid formation-based DRERs is addressed to improve the network resiliency. Ref^8^ proposes a post-event microgrid formation based on the automatic remotely controlled ON/OFF switches and available distributed generators (DGs) to restore critical loads in the distribution network. Furthermore, a resilient microgrid formation based on exact power flow equations with linear characteristics is presented to maximize the total restored load after a natural disaster considering power losses and voltage constraints^9^. However, the authors in^8,9^ don't consider the master–slave capability of DGs in the microgrid formation model. The idea of network reconfiguration based on different DGs with master–slave capacities with single and multiple fault consideration is discussed in^10^. In^11^, a two-stage microgrid formation strategy considers the pre-scheduling of energy storage systems (ESSs) for the first stage and vulnerability analysis for the second stage. However, the consideration of different extreme weather events in the distribution network and the failure period is neglected in^8–11^. Two moderate and heavy damages with different weather scenarios are analyzed through a networked microgrids framework with DRERs formulated in^12^. In^13^, a multi-stage optimization framework of islanded microgrid formation under different weather scenarios is introduced to minimize the total utility cost-based PV-DGs and ESSs, considering budget limitations. Similarly^14^, presents a two-stage multi-step time horizon for restoring critical loads after natural disasters by forming an island microgrid to maximize the total weighted supplied energy. Additionally, an island MG formation via a predictable two-phase natural disaster, windstorm events followed by flooding, in DN aiming to minimize the curtailed energy is presented in^15^. In those studies, the demand response program isn't utilized in the MG formation method after natural disasters.

Considering demand response program for improving the distribution networks resiliency in the microgrid formation process against natural disasters is discussed in^16–24^. A resilience framework of dynamic microgrid formation to restore loads according to their priority after a natural disaster using renewable energies and demand response program is proposed considering emergency budget constraints proposed in^16^. The impact of utilizing internal combustion engine cars is demonstrated in^17^ to enhance the distribution network resiliency through the microgrid formation model after a natural disaster-based demand response program to maximize the prioritized restored load. An optimal microgrid reconfiguration method is formulated for 24-h based on obtaining the best state of interconnecting switches between MGs considering the demand response program to reduce the total operational cost and the total losses^18^. Moreover, in^19^, an incentive-based demand response program is implemented to enhance DN resiliency after natural disasters via a two-stage critical load restoration. However, the load-shedding cost considered the big obstacle after natural disaster occurrence doesn't consider in^16–19^.

Furthermore, a resilience enhancement restorative model-based microgriod formation after extreme events using demand response program with different load characteristics DRERs is presented in^20^ to minimize the load-shedding and the restoration cost. The impact of load-shifting DRP participation to maximize the electricity market profit is addressed in^21^. Authors in^22^, the effect of customer participation and different incentive values in emergency demand response program on microgrid operation discussed to minimize DG operation cost. Additionally, the emergency demand response program is proposed as a smart outage management model to minimize the load-shedding cost in a crisis^23^. In^24^, the microgrid formation after natural disasters is implemented by controlling sectionalizing and tie-line switches within an outage management strategy to enhance the DN resiliency to reduce the restoration cost. A new model of game-based optimization mechanism, namely darts game theory (DGT), is investigated. The darts game theory optimizes the objective function, and the superiority of DGT is indicated when comparing DGT results with other optimization techniques^25^. Ref^26^, a two-stage interactive energy management framework between the distribution network and privately-owned MGs to enhance the resilience of power-water distribution systems considering an incentive demand response program is presented with the objectives of minimizing operation cost and load shedding cost.

Many researchers have been carried out to measure the distribution network resiliency for maintaining a reliable and efficient operation under extreme weather incidents. The resilience metrics can be generally classified into qualitative and quantitative approaches^2,5^. In^12^, a quantitative approach can be classified as 1) Electrical service class, such as total customer energy not served, and 2) Monetary class, such as total outage cost and avoided outage cost. In^27^, another quantitative resiliency metric is formulated, such as recovery time and the time to reach the power balance. The classification of previous resilience studies regarding post-disaster microgrid formation is summarized in Table 1. From the literature review, there are research gaps in the MG formation methods after natural disasters for enhancing distribution network resilience as follows;

1. Many researchers do not consider the cyber-physical coordination of the smart distribution network that enhances bidirectional power and information transfer between the system's agents.
2. Multi-objective optimization problem of the microgrids formation method is not implemented in many researches. Also, the topological constraints of the islanded microgrids formation process based on graph theory to model the physical network are not investigated.
3. Demand response program, as a cost-efficient tool and resilient solution after a natural disaster to avoid outage penalties, is not considered in many researches.
4. Measuring and assessing the resilience of the constructed microgrids after a catastrophic event is not studied in many researches.

**Table 1:** Classification of previous resilience studies regarding post-disaster MG formation.

Refs	Cyber-physical	DRP	Objective			Constraints		Algorithm	Resiliency Metrics
			PL*	LS*	Cost	Topological	Electrical		
^8^		⨯	⨯		⨯			MILP*	⨯
^9^	⨯	⨯	⨯		⨯			MILP*	⨯
^10^	⨯	⨯	⨯		⨯			MISOCP*	⨯
^11^		⨯	⨯	⨯				–	
^12^	⨯	⨯	⨯	⨯	⨯	⨯	⨯	–	
^13^	⨯	⨯		⨯		⨯		NBD*	⨯
^14^	⨯	⨯	⨯		⨯			MILP*	⨯
^15^	⨯	⨯	⨯		⨯	⨯		MILP*	⨯
^16^	⨯		⨯		⨯			MILP*	
^17^	⨯		⨯		⨯			MILP*	⨯
^18^	⨯			⨯		⨯		–	⨯
^19^			⨯		⨯			GBD*	⨯
^20^	⨯		⨯					ε-constraint method	
^21^	⨯		⨯	⨯		⨯		–	⨯
^22^	⨯		⨯	⨯		⨯		MINLP*	⨯
^23^	⨯		⨯			⨯		MILP*	⨯
^24^	⨯		⨯	⨯				–	⨯
Our Paper								DGO	

This paper proposes a multi-objective microgrid formation strategy for the cyber-physical distribution network to enhance resiliency after severe disasters while ensuring topological and electrical constraints. This method is based on the darts game theory approach and emergency demand response program. During natural disasters, the faulty area is isolated from the main supply; hence it's divided into self-sufficient MGs with their DRERs to feed their loads regarding their priority. The DGT optimization technique is applied to obtain optimal formation after a severe disaster. The main objectives are minimizing the power loss, decreasing the load-shedding, and reducing the restoration cost while satisfying the operational constraints of the islanded MGs. The modified IEEE-33 bus system is designed to study the effect of the physical natural disaster and test the proposed microgrid formation method on the DN after extreme weather events. Three case studies are implemented and tested, with and without an EDRP, under a set of scenarios using MATLAB/Script environment. The appropriate resilience metrics are assessed for each case study to evaluate the distribution network resilience to natural disasters.

The main contributions of this paper are summarized as follows;

1. Proposing an optimal formation strategy for microgrids after inevitable catastrophes based on darts game theory, emergency demand response program, and tie-lines for enhancing the distribution network resiliency.
2. Designing a cyber-physical framework of the smart distribution network with its three layers; physical, information, and communication.
3. Using the darts game theory algorithm to solve a multi-objective MG formation to minimize the power loss, load-shed power, and restoration cost to obtain the optimal MG formation and for scenarios' generation and reduction.
4. Comparing the results obtained from the three case studies with and without applying the emergency demand response program and tie-lines to prove the effectiveness of the proposed method.

The remainder of this paper is organized as follows: Section "Cyber-physical framework of the smart distribution network" presents the cyber-physical framework of the smart distribution network. Section "Problem formulation" discusses the problem formulation to formulate the objective functions and the system constraints. Section "Darts game theory (DGT) optimizer" introduces the principles of DGT, its mathematical formulation, and the DGT optimizer for optimal topology. In section "Resiliency metrics formulation", the resiliency metrics are formulated. In section "Test system description", the test system description and the important parameters of the system are presented. In section "Cas studies and results", the three case studies are tested under scenarios. Section "Conclusion" concludes the paper.

PL^*^: Power Loss, LS^*^: Load Shedding, MILP^*^: Mixed Integer Linear Programming, MISOCP^*^: Mixed Integer Second Order Cone Programming, NBD^*^: Nested Bender Decomposition, GBD^*^: Generalized Bender Decomposition, and MINLP^*^: Mixed Integer non-linear Programming.

## Cyber-physical framework of the smart distribution network

Cyber and communication technology transferred the traditional physical network to the cyber-physical network. Furthermore, the power grid may face a blackout due to cyber-attacks and physical disasters^4,8^. So, resilience enhancement against physical and cyber fault disturbances is an essential issue in the load restoration process by altering the network structure, load, and resources. The cyber-physical distribution network includes a complex interaction mechanism of computational and physical devices that adopted modern technologies such as wireless sensor networks (WSN), the internet of things (IoT), smart devices, deeply emerging energy systems, information, and communication system. The proposed cyber-physical distribution network mainly consists of three layers: the physical layer, the information layer, and the communication layer, which are coupled. The communication layer and information layer are called cyber systems collectively. The physical layer includes primary electrical equipment with load consumers and DRERs. The communication layer contains suitable communication infrastructure, equipment, and lines. The information layer is mainly composed of a control center and intelligent interfaces responsible for information collection, monitoring, measurement, metering, and decision control of the entire system. The proposed method focuses on physical natural disasters such as hurricanes, earthquakes, and floods and the cyber network is considered a cloud communication network that allows the DN operator to collect information about predicting sudden disasters, consumer behavior, and energy production. This in turn is useful for this work when applying a multi-step emergency demand response program where the DN operator signs contracts with participants before the disaster to minimize the outage cost. The collected information is sent to the physical network through the communication network. The role of information layer and its serious impact on resiliency is cleared when hampering the data process, the DN operator may receive misleading feedback, and its result is power outage^11^. The cyber layer is not entered in the microgrid formation and optimization problem.

**Figure 2:**
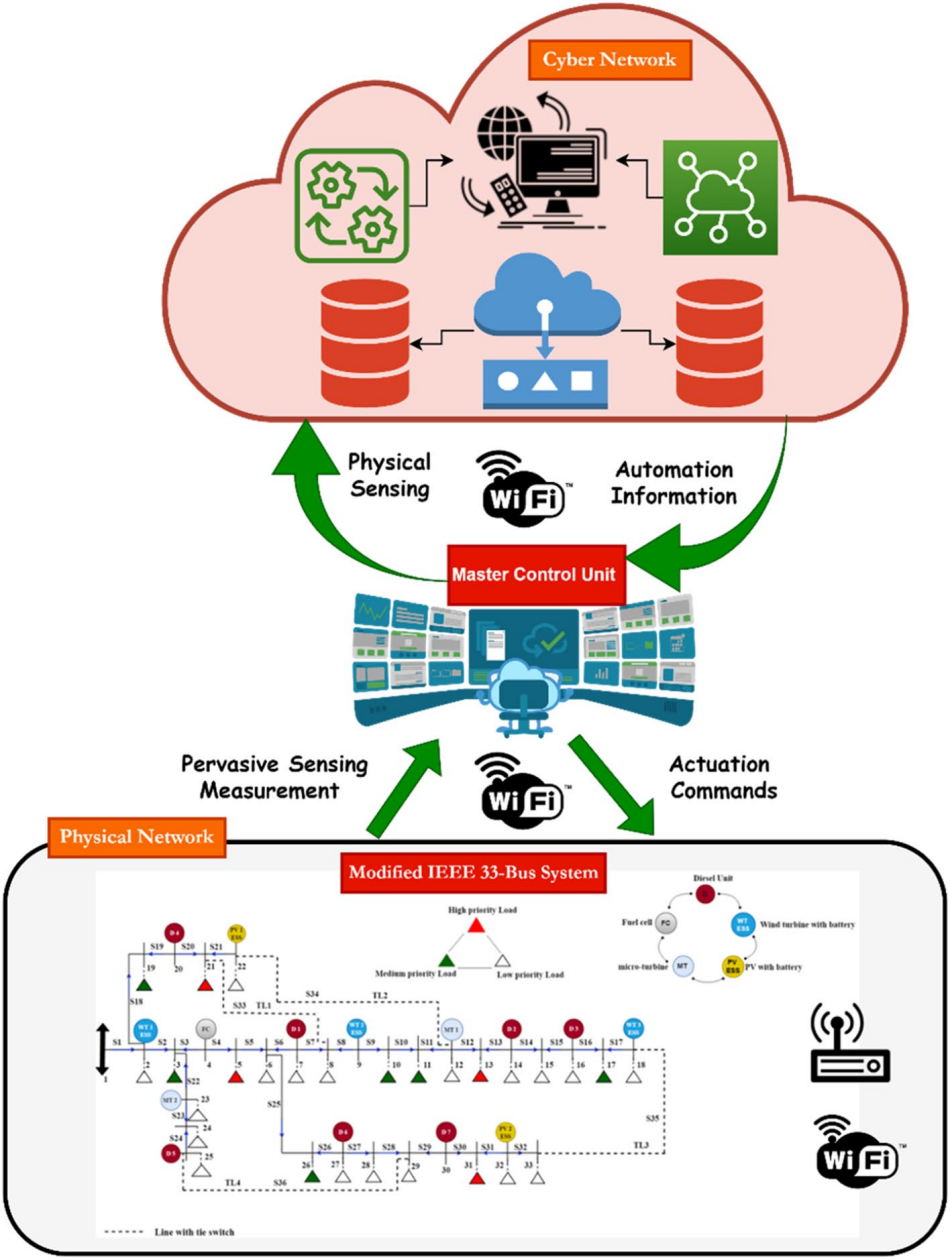
Structure of cyber-physical distribution network.

The structure of the cyber-physical distribution network is shown in Fig. 2. The theoretical model of cyber-physical distribution network is associated with modeling the incidence matrix and analyzing network topology. According to a network graph theory and matrix operation model, the model of a physical information network is constructed as follows^2^;

1 $$\left\{\begin{array}{l}G=\left(\mathrm{V},\mathrm{E},{\mathrm{A}}_{\mathrm{c}}\right)\\ V=\left\{{\mathrm{v}}_{1},{\mathrm{v}}_{2},\dots ,{\mathrm{v}}_{\mathrm{p}}\right\}\\ E=\left\{{\mathrm{E}}_{\mathrm{qj}}\right\}\\ {\mathrm{A}}_{\mathrm{c}}=\{{\mathrm{A}}_{\mathrm{c},\mathrm{qj}}\}\end{array}\right.$$

where, $\mathrm{V}=\left\{{\mathrm{v}}_{1},{\mathrm{v}}_{2},\dots ,{\mathrm{v}}_{\mathrm{o}}\right\}$ is the set of cyber-physical DN nodes. $\mathrm{E}=\left\{{\mathrm{E}}_{\mathrm{qj}}\right\}$ is the connection matrix between two nodes, ${\mathrm{E}}_{\mathrm{qj}}$ is the connection state between node $\mathrm{q}$ and node $\mathrm{j}$, when there's a link between node ${\text{q}}$ and node ${\text{j}},$ ${\text{E}}_{{{\text{qj}}}} { } = 1$, otherwise ${\text{E}}_{{{\text{qj}}}} { } = 0$. ${\text{ A}}_{{\text{c}}} = \left\{ {{\text{A}}_{{{\text{c}},{\text{qj}}}} } \right\}$ is the incidence matrix between the physical network and the cyber network, ${\text{A}}_{{{\text{c}},{\text{qj}}}}$ is the connection status between the physical network node $q$ and the cyber network node ${\text{j}}$. When there's a connection between node ${\text{i}}$ and node ${\text{ j}},$ ${\text{A}}_{{{\text{c}},{\text{qj}}}} { } = 1$, Otherwise ${\text{A}}_{{{\text{c}},{\text{qj}}}} = 0$.

## Problem formulation

When a cyber-physical DN operator faces single or multiple faults after natural disasters, the distribution network is isolated from the main utility grid, and some switches are opened. Several unsupplied electrical islands can be formed. The approved solution in this situation is to divide a distribution network into some self-supplied microgrids with their DRERs to feed loads according to their priorities. The DN operator might be forced to pay outage penalties after natural disasters due to applying for the costly load-shedding program in the distribution network. The emerging of the demand response program is one of the cost-efficient and resilient solutions to avoid these outage penalties and enhance the resiliency of the cyber-physical distribution network. The network topology can be reconfigured to restore more loads by implementing tie switches, meaning that the load at one feeder can be transferred to another feeder in the microgrid formation model.

In this paper, the objective functions and the system constraints of the cyber-physical DN-MG formation method based on the DGT technique and multi-step EDRP after a natural disaster are formulated as follows,

### Objective functions

The objectives are minimizing total power loss, load shedding, and total restoration cost. The objective function can be expressed as follows^20,28^;

#### Network power losses

2 $$ min \left( {P_{{loss_{t} }} } \right) = min\left( {\mathop \sum \limits_{s} \left[ {\mathop \sum \limits_{mg = 1}^{{N_{mg} }} \mathop \sum \limits_{{\left( {n,m} \right) \in N_{bus} }} r_{n,m} I_{n,m}^{2} } \right]} \right) $$

where $$P_{{loss_{t} }}$$ is the total power losses of formable MGs at time $$t$$.$$ N_{mg}$$ and $$ N_{bus}$$ represent a set of MGs and a set of buses, and $$r_{n,m}$$ and $$I_{n,m}$$ represent resistance and current between bus $$ n $$ and bus $$ m$$ , $$s$$ represents a set of scenarios^28^.

#### Network load shedding

3 $$ min\left( {P_{{shedd_{t} }} } \right) = min \left( {\mathop \sum \limits_{s} \left[ {\mathop \sum \limits_{b = 1}^{{N_{bus} }} \mathop \sum \limits_{t} PG_{b,mg,s,t} - \mathop \sum \limits_{b = 1}^{{N_{bus} }} \mathop \sum \limits_{mg = 1}^{{N_{mg} }} \mathop \sum \limits_{t} \omega_{b,s,t}^{pr} *PD_{b,mg,s,t} } \right]} \right) $$

where $$P_{{shedd_{t} }}$$ is the total load shedding of constructed $$mg$$th MGs. $$N_{bus}$$ represents a set of $$b$$th buses. $$PG_{b,mg,s,t}$$ and $$PD_{b,mg,s,t} $$ represents active power generated and active power demand at bus $$ b$$.$$ \omega_{b,s,t}^{pr}$$ represents the priority of load ^20^.

#### Network restoration cost

4 $$ min(C_{{rest_{t} }} ) = min\left( {\mathop \sum \limits_{s} \left[ {\mathop \sum \limits_{t} C_{g,s,t} + \mathop \sum \limits_{t} C_{op,s,t} + \mathop \sum \limits_{t} C_{EDRP,s,t} } \right] } \right) $$

5 $$ C_{g,s,t} = \mathop \sum \limits_{mg = 1}^{{N_{mg} }} P_{g,mg,s,t} *\tau_{g} , \forall g,s,t $$

where, $$C_{{rest_{t} }}$$ is the total restoration cost of formable $$mg^{th}$$ MGs. $$C_{g,s,t}$$ represents the operational cost of $$g^{th }$$ DRERs.$$ C_{op,s,t}$$ is the outage penalty cost due to natural disasters.$$ C_{EDRP,s,t}$$ is the multi-step EDRP cost. $$P_{g,mg,s,t}$$ represents active power output. $$\tau_{g}$$ represents the operational cost coefficient price ($/kW)^20^.

The following constraints should be satisfied for MG formation under natural disasters to meet the required objectives.

### Topological constraints

Graph theory is implemented in this model to represent the topological constraints of the islanded post-event MG formation method. The set of nodes that can be considered master units is considered by $N^{master}$, and the set of nodes that are connected to DGs is denoted by $N^{dg}$. The topological constraints can be modeled as follows^17,19,20^. To guarantee that each node can belong to one of the formed MGs or it none of them, Eq. (6) can be represented as follows to model this concept^23^,

6 $$ \mathop \sum \limits_{mg = 1}^{{N_{mg} }} \alpha_{n,mg} \le 1, \forall n \in N_{bus} $$

where $\alpha_{n,mg}$ represents binary variable (1 if bus $n$ belongs to MG, 0 otherwise).

- Node $n$ can be connected to MG if the $mg^{th}$ member of $N^{master} \left( {r = N^{master} \left( {mg} \right)} \right)$ and $\alpha_{r,mg} = 1$ selected as the root node. The root node constraints are expressed as follows^17^,
  7 $$ \alpha_{n,mg} \le \alpha_{r,mg} ,r = N^{master} \left( {mg} \right),\forall n \in N_{bus} $$
  where $\partial_{l}$,$\vartheta_{l}$ are the set of start and end points of line $l$ . $\beta_{l,mg}$ represents binary variable (1 if line $l$ in MG is active, 0 otherwise). $L$ represents a set of lines $.$
- During the formation of islanded MG, if the two sides of a line are not located on the same MG, the binary variable status of the line must be zero. This can be formulated as follows^17^,
  8 $$ Line_{l} = \mathop \sum \limits_{mg = 1}^{{N_{mg} }} \beta_{l,mg} , \forall l \in L $$
  9 $$ \beta_{l,mg} \le \mathop \sum \limits_{{n \in \partial_{l} }} \alpha_{n,mg} , \forall n \in N_{bus} $$
  10 $$ \beta_{l,mg} \le \mathop \sum \limits_{{n \in \vartheta_{l} }} \alpha_{n,mg} , \forall n \in N_{bus} $$
  11 $$ \beta_{l,mg} \ge \mathop \sum \limits_{{n \in \vartheta_{l} }} \alpha_{n,mg} + \mathop \sum \limits_{{n \in \partial_{l} }} \alpha_{n,mg} - 1, \forall n \in N_{bus} $$
  where $\partial_{l}$, $\vartheta_{l}$ are the set of start and end points of line $l$ . $\beta_{l,mg}$ represents binary variable (1 if line $l$ in MG is active, 0 otherwise). $L$ represents a set of lines $.$

- The line status and the damaged bus status are indicated in (12)-(13). Also, the switch status can be represented in (14) to indicate that there are no remote-control switches on all lines in the DN^20^,
  12 $$ \beta_{l,mg} \le Line_{l}^{initial} ,\forall l \in L $$
  13 $$ \alpha_{n,mg} \le Bus_{n}^{initial} ,\forall n \in N_{bus} $$
  14 $$ \alpha_{n,mg} \le \alpha_{r,mg} ,\forall n,r \in N_{bus} ,\forall l \in L and \rho_{l,n,r} = 1 $$
  where $Line_{l}^{initial}$ , $Bus_{n}^{initial}$ represents the initial status of lines and buses. $\rho_{l,n,r}$ represents binary variable (if there is a line between bus $n$ and $r$ , 0 otherwise).

- To guarantee the radiality, spanning tree constraints^19^ for each formed MG are formulated in (15)-(17)
  15 $$ \alpha_{nm} + \alpha_{mn} = \beta_{nm} \forall \left( {n,m} \right) \in E $$
  16 $$ \mathop \sum \limits_{m \in \varphi \left( n \right)} \alpha_{nm} = 1 \forall n \in N_{t} \backslash r $$
  17 $$ \alpha_{nm} = 0 \forall n \in r,m \in \varphi \left( n \right) $$
  where $\alpha_{nm}$ binary variable indicating the parents of nodes, if node $m$ is a parent of node $n$, $\alpha_{nm} = 1$, $\beta_{nm}$ represent the status of line $nm$ . $N_{t}$ set of total nodes in the DN. $E$ set of all available lines in the post-disaster DN. $\varphi \left( n \right)$ set of neighboring nodes of the node $n$. $r$ is the root node of the spanning tree.

### Electrical constraint

#### ***DRERs constraints***:

18 $$ PG_{g,mg,s,t}^{min} \le PG_{g,mg,s,t} \le PG_{g,mg,s,t}^{max} $$

19 $$ QG_{g,mg,s,t}^{min} \le QG_{g,mg,s,t} \le QG_{g,mg,s,t}^{max} $$

where $$PG_{g,mg,s,t} ,QG_{g,mg,s,t}$$ represent active and reactive power generated, $$PG_{g,mg,s,t }^{min}$$ and $$PG_{g,mg,s,t}^{max}$$ represent minimum and maximum allowed active output, $$QG_{g,mg,s,t}^{min}$$ and $$ QG_{g,mg,s,t}^{max}$$ represent the minimum and maximum allowed reactive output^11^.

#### Energy storage system (ESS) constraints

20 $$ 0 \le P_{e,mg,s,t}^{ch} \le P_{e,mg,s,t}^{ch,max} \mu_{e,t}^{ch} $$

21 $$ 0 \le P_{e,mg,s,t}^{dch} \le P_{e,mg,s,t}^{dch,max} \mu_{e,t}^{dch} $$

22 $$ \mu_{e,mg,s,t}^{ch} + \mu_{e,mg,s,t}^{dch} \le 1 $$

The state of charge (SoC) of ESS and its limits are presented as follows,

23 $$ SoC_{e,mg,s,t} = SoC_{e,mg,s,t - 1} + \frac{1}{{EC_{e} }}\left( {\mu^{ch} P_{e,mg,s,t}^{ch} - \frac{1}{{\mu^{dch} }}P_{e,mg,s,t}^{dch} } \right) $$

24 $$ SoC_{e}^{min} \le SoC_{{P_{e,mg,s,t}^{dch} }} \le SoC_{e}^{max} $$

where $P_{e,mg,s,t}^{ch} ,P_{e,mg,s,t}^{dch}$ represent the charging and discharging power of the $e^{th}$ ESS.${\text{ SoC}}_{{\text{e}}}^{{{\text{min}}}} ,$ ${\text{SoC}}_{{\text{e}}}^{{{\text{max}}}}$ Represent minimum and maximum state of charge, ${\text{EC}}_{{\text{e}}}$ represents the capacity of ESS and ${{ \upmu }}^{{{\text{ch}}}} ,{{ \upmu }}^{{{\text{dch}}}}$ represent the charging and discharging efficiency of ESS^29^.

#### Power balance constraints

25 $$ \mathop \sum \limits_{mg = 1}^{{N_{mg} }} \left( {PG_{n,mg,s,t} + P_{e,mg,s,t}^{dch} - P_{e,mg,s,t}^{ch} - PD_{n,mg,s,t} } \right) = \mathop \sum \limits_{mg = 1}^{{N_{mg} }} P_{nm} $$

26 $$ \mathop \sum \limits_{mg = 1}^{{N_{mg} }} \left( {QG_{n,mg,s,t} - QD_{n,mg,s,t} } \right) = \mathop \sum \limits_{mg = 1}^{{N_{mg} }} Q_{nm} $$

where $$P_{nm}$$ , $$Q_{nm}$$ represent the active and reactive power flow in branch $$nm$$. $$PG_{n,mg,s,t }$$, $$QG_{n,mg,s,t}$$ represent active and reactive power generated by $$n$$ bus, $$PD_{n,mg,s,t}$$ , $$QD_{n,mg,s,t} $$ represent active and reactive power demand at bus $$n$$^20^.

#### Power flow constraints

27 $$ P_{n,m}^{flow} = P_{m}^{inf} + \mathop \sum \limits_{b:m \to b} P_{m,b}^{flow} + r_{n,m} I_{n,m}^{2} , \quad \forall \left( {n,m} \right) \in N_{bus} $$

28 $$ Q_{n,m}^{flow} = Q_{m}^{inf} + \mathop \sum \limits_{b:m \to b} Q_{m,b}^{flow} + x_{n,m} I_{n,m}^{2} , \quad \forall \left( {n,m} \right) \in N_{bus} $$

where $$P_{n,m}^{flow}$$ and $$Q_{n,m}^{flow}$$ represent active and reactive power flows between bus $$n$$ and $$m $$. $$P_{m}^{inf}$$ and $$Q_{m}^{inf}$$ represent the inflexible active and reactive power. $$r_{n,m}$$ and $$x_{n,m}$$ represent resistance and reactance. $$I$$ represents the current between buses^28^.

#### Bus voltage constraints

29 $$ V_{m}^{2} = V_{n}^{2} - 2\left( {r_{n,m} P_{n,m}^{flow} + x_{n,m} Q_{n,m}^{flow} } \right) + \left( {r_{n,m}^{2} + x_{n,m}^{2} } \right)I_{n,m}^{2} , \forall \left( {n,m} \right) \in N_{bus} $$

30 $$ \left| V \right|_{m}^{min} \le \left| V \right|_{m} \le \left| V \right|_{m}^{max} ,\quad \forall \left( {n,m} \right) \in N_{bus} $$

where $V_{n}$ represents the voltage at bus $n$ ,$\left| V \right|_{m}^{min}$, $\left| V \right|_{m}^{max}$ represent minimum and maximum voltage magnitude at bus $m$, $\left| V \right|_{m}$ represents the voltage magnitude at bus $m$^28^.

#### Emergency demand response program constraints

31 $$ EDRP_{d,mg,s,t}^{min}  \le P_{EDRPd,mg,s,t}^{c} \le EDRP_{d,mg,s,t}^{c} $$

32 $$ P_{{EDRP_{d,mg,s,t} }} = \mathop \sum \limits_{c = 1}^{C} P_{EDRPd,mg,s,t}^{c} $$

33 $$ EDRP_{d,mg,s,t}^{min} = {\text{ k}}^{{{\text{min}}}} PD_{n,mg,s,t} { },\quad \forall n \in N_{bus} $$

34 $$ EDRP_{d,mg,s,t}^{c} = {\text{ k}}^{c} PD_{n,mg,s,t} ,\quad \forall n \in N_{bus} { },{\text{ c}} = 1,2, \ldots {\text{C}} $$

where $$ P_{EDRPd,mg,s,t}^{c}$$ represent offered load reduction of $$d^{th}$$ EDRP blocks. $$EDRP_{d,mg,s,t}^{c} ,EDRP_{d,mg,s,t}^{min}$$ represent maximum load reduction of $$c^{th}$$ step of EDRP, minimum load reduction of EDRP.$${ }k^{c} { }and{ }k^{min}$$ represent the coefficient of the adjusted demand reduction with a step of EDRP, the coefficient of the minimum allowed demand reduction^28^.

To solve this problem, an optimization method based on DGT is proposed to achieve the objective function by considering the system constraints.

## Darts game theory (DGT) optimizer

This paper uses darts game theory to solve the optimization problem to obtain an optimal post-event microgrid formation for the cyber-physical distribution network after extreme disasters. It aims to achieve the objectives by considering the aforementioned system constraints, which are used to reduce the number of scenarios generated for MG formation. The darts game theory is a sport that consists of a dartboard, darts, and darts players with their goals. The dartboard has 82 areas with different points, and the DGT key idea is when players try to get possible points in their throws toward the game board. The throw scoring in the inner bull has 50 points, and the outer bull has 25 points. When the darts hit the narrow inner ring, the score is tripled. However, the score doubles if the throw hits the narrow outer ring. The dashboard's center is considered the game's fifth-highest scoring area. The angle intervals and the areas of the different sectors in the game are presented in^25^. Such as with other optimization techniques, the DGT approach can be modeled to get the highest score or the optimal solution which depends on a set of equations and parameters as represented in the following subsections.

DGT algorithm is developed to find possible solutions and choose the optimal one. By assumption, each player has three throws to build its score matrix, $$S$$. At each throw for each player, the fitness functions and their variable values are determined, and then determine best and worst fitness functions and their best and worst variable values. The next step is to calculate the normalized value of fitness functions $$ Ft^{n}$$ And the probability function $$P_{i}$$ of $$i^{th}$$ player. Finally, to find the winner, the points for each player $${\text{S}}_{{\text{i}}}^{{\text{n}}}$$ is calculated in every throw for each player. The status of each player and his variable values are renewed. By assumption, the number of iterations in this game is 100 times.

DGT optimizer also can be used for decreasing the number of scenarios generated within the microgrid formation process to a tractable number regarding the objectives concerning the set of constraints. The score function is calculated for each scenario at each iteration of the solution procedure. The scenario with the lowest probability and score to other scenarios is removed. This procedure is repeated until the desired number of recorded scenarios is attained.
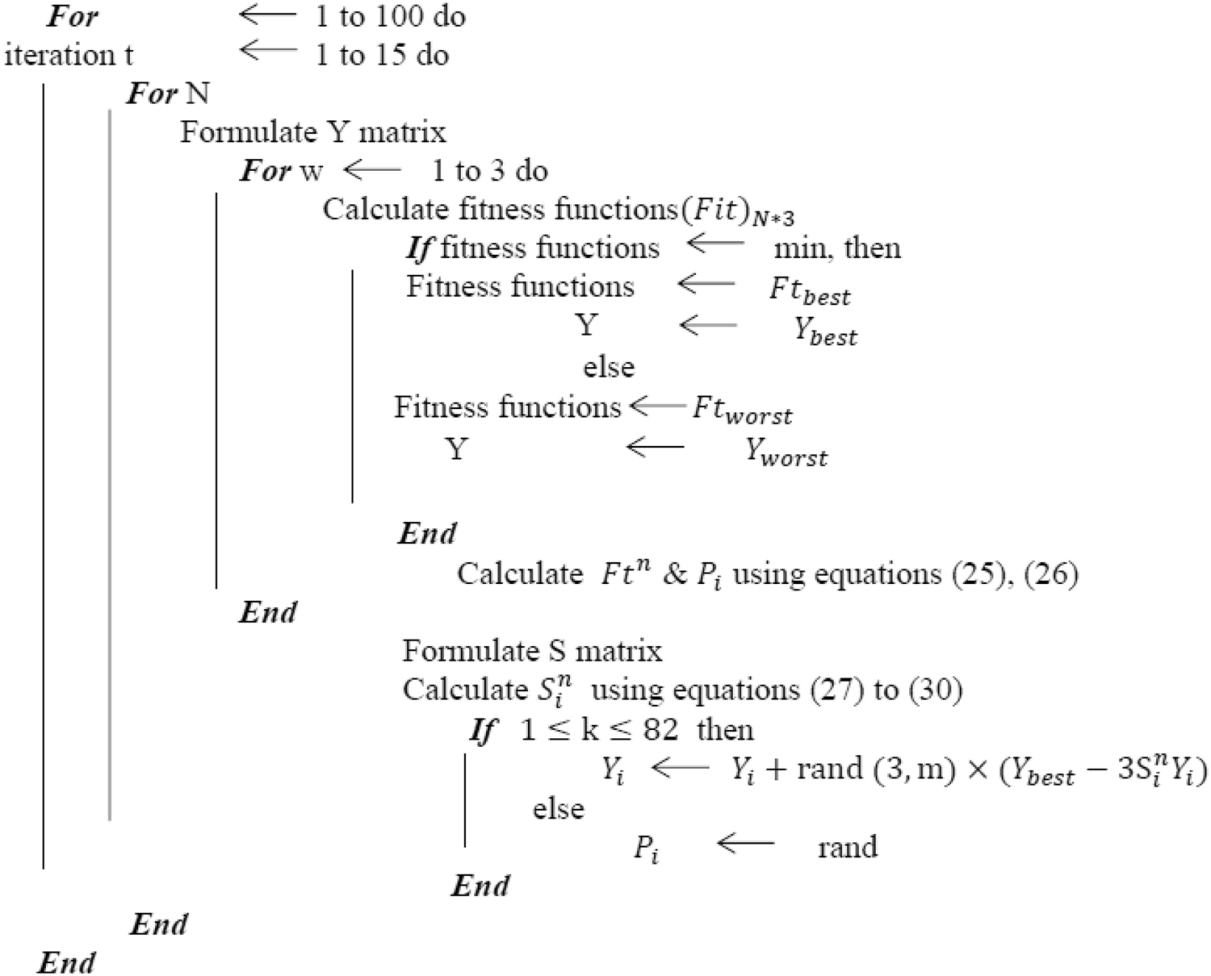


## Resiliency metrics formulation

The system resiliency of islanded microgrids with distributed renewable energy resources can be measured to study the impact of power interruption on customers and electricity utilities. The formulation of these metrics is represented as follows^11,12,20^.

### Total amount of energy not served (kWh)

42 $$ ENS = \mathop \sum \limits_{t = 1}^{v} E_{mg}^{shedd} \left( t \right) $$

where $E_{mg}^{shedd} \left( t \right)$ is the total amount of energy not served per MG $mg$ for the event $v$ .

### Total loss of utility revenue ($)

43 $$ C_{LOUR,s} = C_{en} * \mathop \sum \limits_{t = 1}^{v} E_{mg}^{shedd} \left( t \right) $$

where $${ }C_{LOUR,s}$$ is the total loss of utility revenue per scenario $$s$$ ($), and $$C_{en}$$ is the cost of energy ($/kWh).

### Total outage penalty cost ($)

44 $$ C_{op,s} = C_{opf} *\mathop \sum \limits_{t = 1}^{v} E_{mg}^{shedd} \left( t \right) $$

where $$ C_{op,s}$$ is the total outage penalty cost ($), and $$ C_{opf}$$ is the outage penalty factor the DN operator forces to pay when applying the load-shedding program ($/kW-hr).

### Total avoided outage cost

45 $$ C_{avd,s} = C_{op,s,before EDRP} - C_{op,s,after EDRP} $$

where $$ C_{avd,s}$$ is the total avoided outage cost, ($), $$C_{out,s,before EDRP}$$ is the total outage penalty cost before applying EDRP. $$C_{op,s,after EDRP}$$ is the total outage penalty cost after applying EDRP.

### Resilience index (RI)

46 $$ RI_{s}^{Net} = \frac{{\mathop \sum \nolimits_{b = 1}^{{N_{bus} }} \mathop \sum \nolimits_{mg = 1}^{{N_{mg} }} \mathop \sum \nolimits_{t} w_{b,s,t}^{pr} * PD_{b,mg,s,t}^{recovered} }}{{\mathop \sum \nolimits_{b = 1}^{{N_{bus} }} \mathop \sum \nolimits_{t} PD_{b,s,t} }} $$

where $$ RI_{s}^{Net}$$ is the network resilience index, $$PD_{b,mg,s,t}^{recovered}$$ is the recovered active power demand at bus $$ b$$.

## Test system description

**Figure 3:**
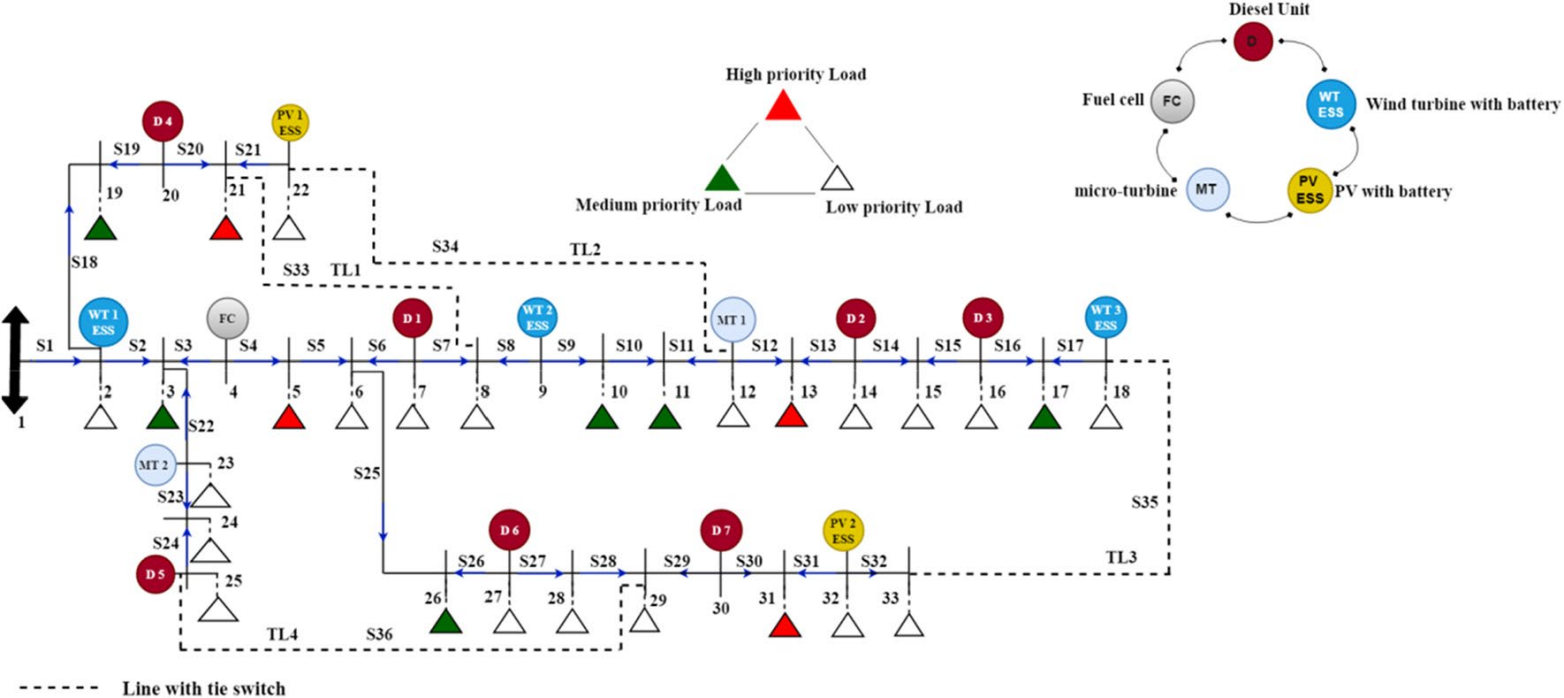
Modified IEEE 33-bus test system.

In this paper, the modified IEEE 33 bus system structure shown in Fig. 3 is implemented in^17^ using a MATLAB/Script environment to study the effect of the physical natural disaster and test the proposed post-event microgrid formation method on the distribution network after extreme weather events. The system consists of 32 sectionalizing switches and four tie line switches that can be controlled by the cyber network remotely, a combination of fifteen DRERs which are divided into five types of renewable energy with ESS (two photo-voltaic units (PV) at buses 22, and 32, three wind turbines (WTs) at buses 2, 9, and 18, seven diesel generators at buses 7, 14, 16, 20, 25, 27, and 30, two micro-turbine (MT) at buses 12, and 23, one fuel cell (FC) at bus 4, and 28 load points with different priorities. The base power and voltage of the system are 100 MVA and 11 kV, respectively. The total active and reactive power consumption are 4.61 Mw and 2.15 MVAr, respectively. The generation capacity of WT with ESS, PV with ESS, and MT is 0.2 MW, while the diesel unit and FC generation capacity is 0.25 MW. The maximum generation capacity of all DRERs is 0.3 MW. The ESSs capacity, charging, discharging rates, efficiencies, and initial depth of discharge is 0.2 MWh, 0.2 MW, 85%, and 33%, respectively.

**Figure 4:**
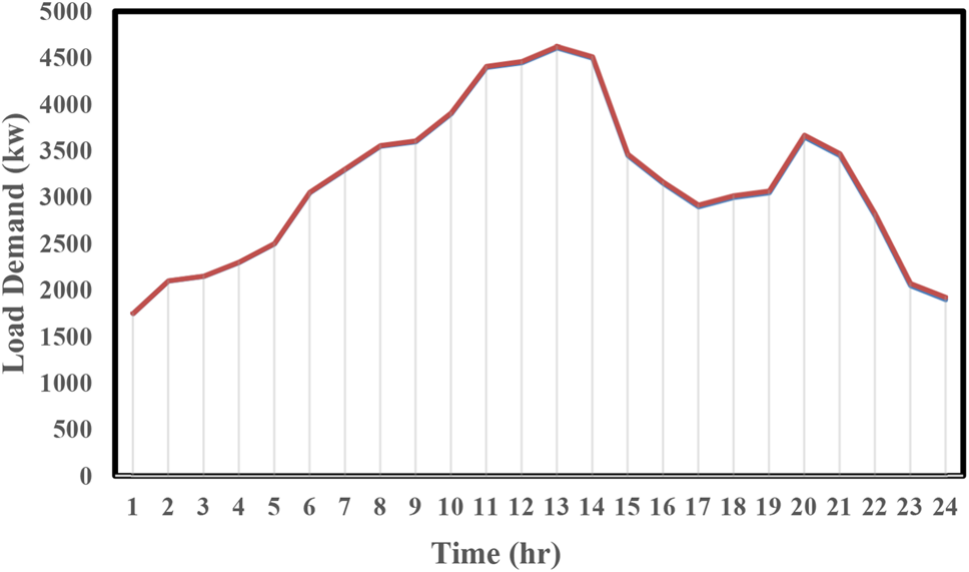
Hourly load demand profile for a sample day^11^.

The hourly load demand profile for a sample day of distribution network is shown in Fig. 4^11^. The operation cost coefficient of the diesel unit, FC, MT, PV, WT, and ESS is 0.08, 0.07, 0.06, 0.01, 0.01, and 0.01 $/kWh, respectively^20^. In the proposed model, there are three types of loads, low-priority, medium, and high or critical-priority loads, and their priority factor is assumed to be 0.1, 10, and 100, respectively^19^. Three case studies are considered in the following section to show the effect of implementing emergency demand response program and tie-lines on the proposed model.

## Cas studies and results

This paper applies three case studies to test the proposed microgrid formation strategy after a drastic incident. The three cases are as follows;

1. Without applying EDRP
2. With applying EDRP
3. With applying EDRP and tie switches

In the three case studies, a single fault is assumed to be occurred in the test system due to extreme events, and the fault location is shown with a red cross, which isolates the distribution network from the main utility supply; hence the distribution network is operated in islanded mode with outage occurring over the one hour following the natural disaster event.

Applying the DGT optimizer for scenario reduction, the best five scenarios are chosen concerning the best fitness values. The microgrid formation strategy for enhancing the resiliency of the distribution network is applied and studied for the five scenarios for each case study.

### First case: without applying EDRP

In this case, the performance of five scenarios is analyzed without emergency demand response program to achieve the objectives under a set of constraints. Each microgrid is a separate system with its adjacency matrix and DRERs responsible for feeding its loads. When the system runs, power system studies such as power flow and loss calculations are applied to execute the objectives, and values of operational constraints are calculated. In many cases, the generated output power is less than the demand power, and therefore the load-shedding program is applied, and this forces the DN operator to pay a penalty cost called outage penalty cost (${\text{C}}_{{{\text{op}},{\text{s}}}}$) to customers. The penalty factor for load shedding programs for each load is assumed to be between 10 and 14 USD/kWh according to the load priority^23^. In this regard, the penalty factor is assumed to be equal to 10, 12, and 14 USD/kWh for low, medium, and high-priority loads, respectively. The DN operator strategy of applying the load-shedding is started with low-priority loads, then medium-priority loads, and finally high priority loads to reduce outage penalty cost as much as possible. It's noted that this case is considered the base case, so the total avoided outage cost metric is not calculated. Table 2 represents the objectives and resiliency metrics calculations for each scenario.

**Table 2:** Summary of the objectives and resiliency metrics for the five scenarios without EDRP.

	Scenario 1	Scenario 2	Scenario 3	Scenario 4	Scenario 5
Total power losses (MW)	0.016	0.015	0.016	0.015	0.016
Total load shedding (MW)	1.01	1.19	0.982	0.903	0.931
Total restoration cost ($)	10,307.5	12,713.1	10,029.74	9766.06	9803.82
Total amount of energy not-served (kWh)	1010	1190	982	903	931
Total loss of utility revenue ($)	45.45	55.1	44.19	41.93	42.59
Total outage cost ($)	10,100	12,520	9820	9550	9590
Resiliency Index	0.7809	0.7418	0.7869	0.8041	0.7980

**Figure 5:**
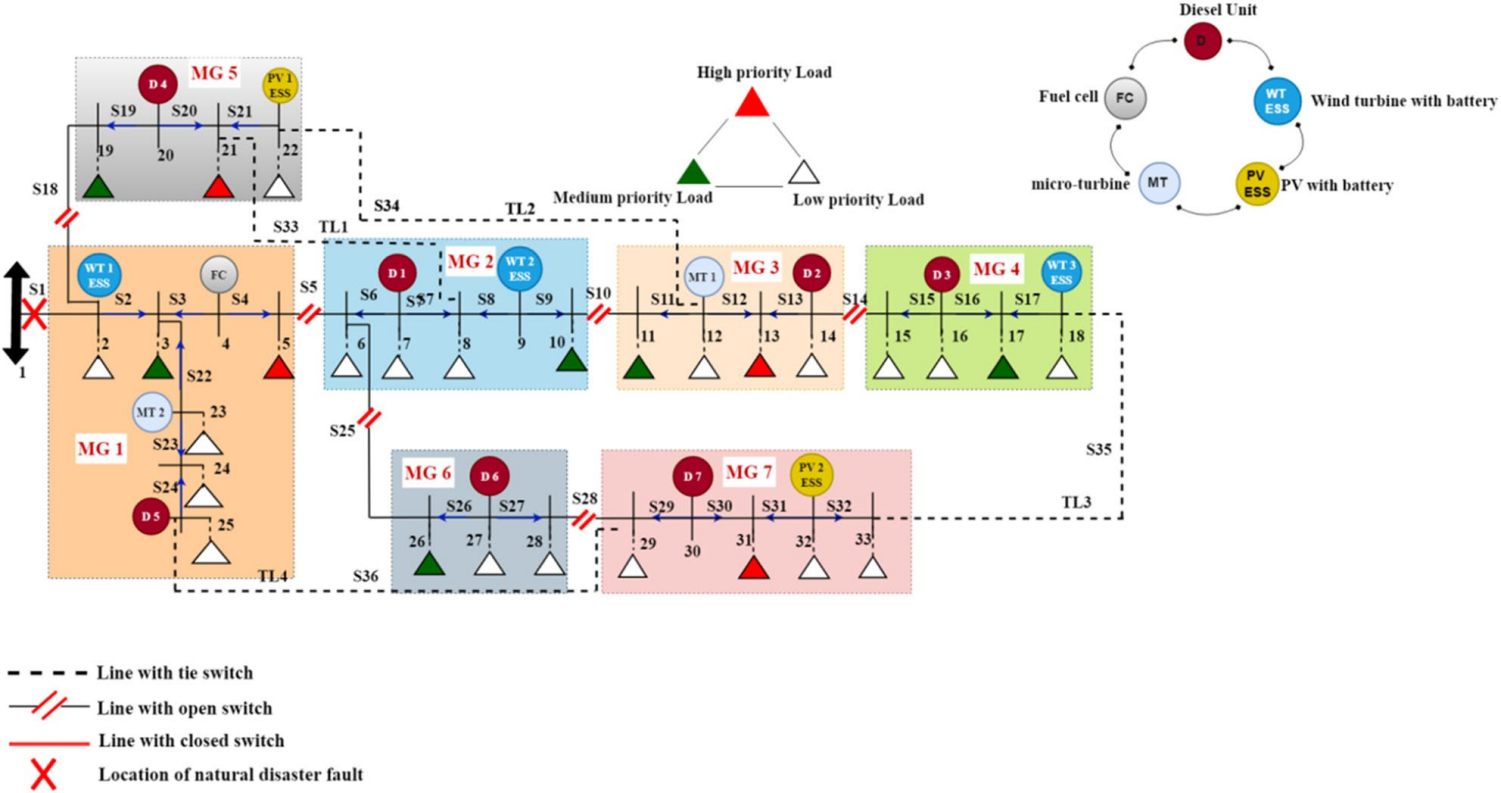
The optimal MG formation scenario without EDRP.

From Table 2, the scenario with the best objective functions and more resilient out of all scenarios is scenario 4, so it's the best solution for the microgrid formation for the first case study, as shown in Fig. 5.

### Second case: applying EDRP

**Figure 6:**
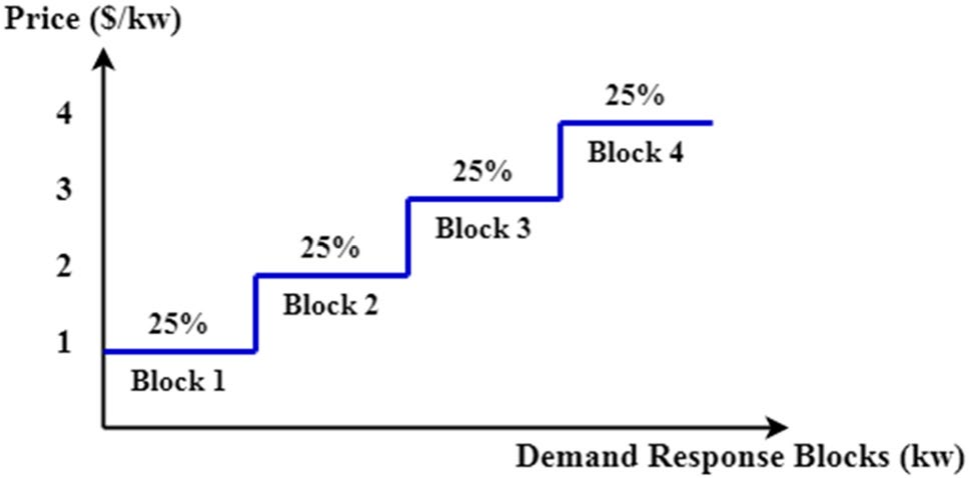
The step cost of EDRP program in the MG formation model.

In this case, the same five scenarios of the first case study are implemented to achieve better performance by applying emergency demand response program. This paper pays the low-priority loads of each microgrid for five scenarios within a multi-step emergency demand response program program. The demand response program price increases with each step, meaning that the first step has the lowest price. These incentive payments are much lower than the outage penalty cost when applying for the load-shedding program. In this paper, the low-priority loads in each MG for the five scenarios are assumed to offer four DRP blocks, each of which is 25% of the total loads, and the DN operator can use all the DRP blocks of the loads if needed. The EDRP step cost is 1, 2, 3, and 4 $/kW, respectively. The proposed emergency demand response program, implemented in the microgrid formation method, and the step cost are shown in Fig. 6^23^. Table 3 represents the objectives and resiliency metrics calculations for each scenario.

**Table 3:** Summary of the objectives and resiliency metrics for the five scenarios with EDRP.

	Scenario 1	Scenario 2	Scenario 3	Scenario 4	Scenario 5
Total power losses (MW)	0.016	0.014	0.016	0.014	0.014
Total load shedding (MW)	0.029	0.333	0.021	0.277	0.166
Total restoration cost ($)	2424.14	5241.5	2373.88	4361.18	3580.66
Total amount of energy not-served (kwh)	29	333	21	277	166
Total loss of utility revenue ($)	1.305	14.985	0.945	12.465	7.47
Total outage cost ($)	2220	5055	2167.5	4150	3375
Total avoided outage cost ($)	7880	7465	7652.5	5400	6215
Resiliency Index	0.9899	0.9109	0.9941	0.9293	0.9556

**Figure 7:**
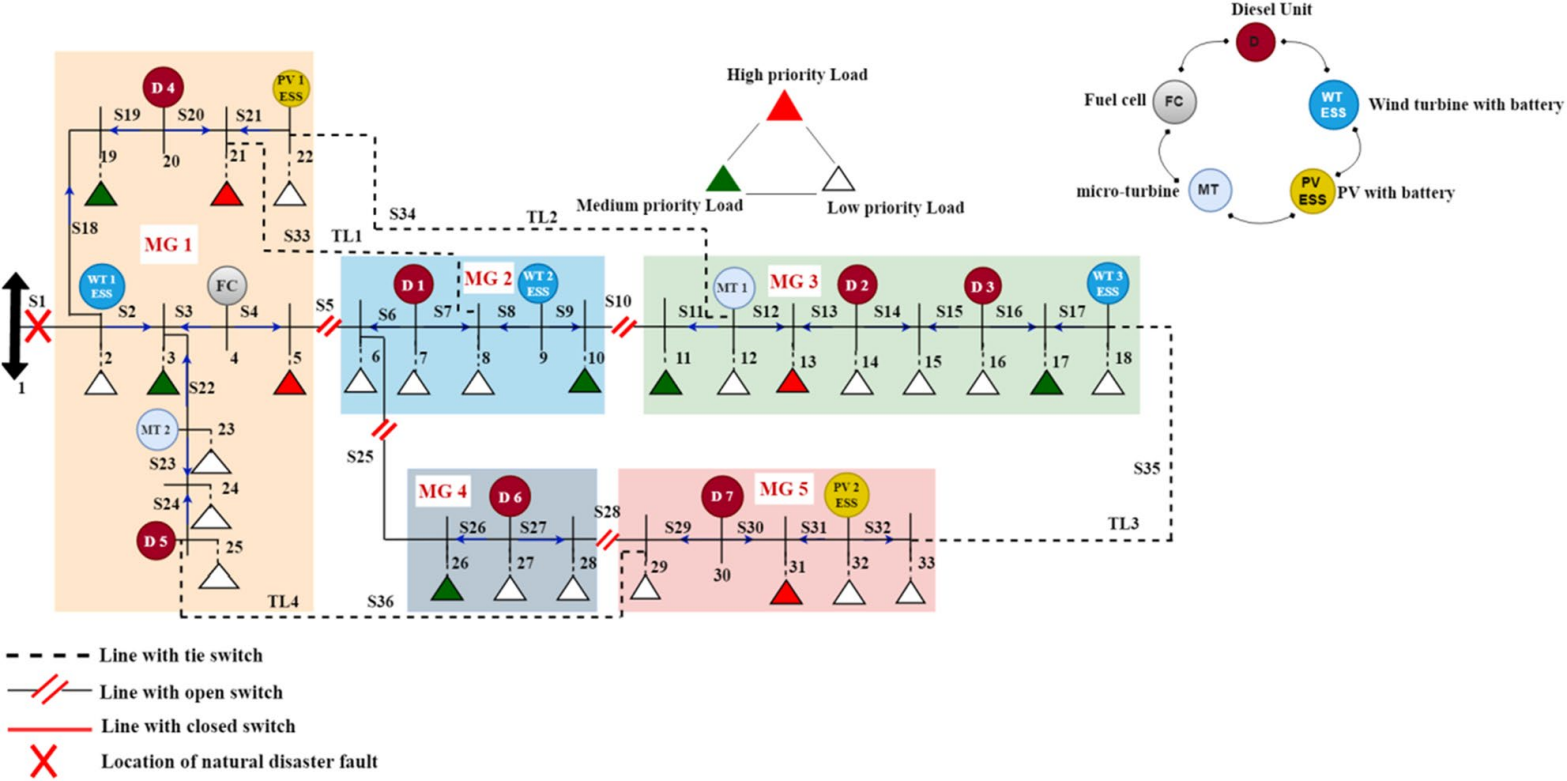
The optimal MG formation scenario with EDRP.

From Table 3, concerning the load shedding and restoration cost as the most important objectives, the scenario with the best performance and more resilience is scenario 3, shown in Fig. 7.

From Table 3, concerning the load shedding and restoration cost as the most important objectives, the scenario with the best performance and more resilience is scenario 3, shown in Fig. 4.

### Third case: applying EDRP and Tie-line switches

Normally open tie-lines in the cyber-physical distribution network can be exploited as an efficient tool for improving network resiliency by applying the emergency demand response program to attain better performance than the first case and the second case study. The location of the selected Tie-lines is according to^11,19^. After natural disasters, the network's topology may be reconfigured by closing tie switches to restore the islanded loads by transferring them to another feeder.

Similarly, applying the DGT optimizer for scenario reduction, the best five scenarios are chosen and analyzed concerning the best fitness values. Table 4 represents the objectives and resiliency metrics calculations for each scenario.

**Table 4:** Summary of the objectives and resiliency metrics for the five scenarios with EDRP and Tie-line.

	Scenario 1	Scenario 2	Scenario 3	Scenario 4	Scenario 5
Total power losses (MW)	0.013	0.014	0.016	0.018	0.015
Total load shedding (MW)	0.014	0.372	0.018	0.019	0.103
Total restoration cost ($)	1199.52	5528.54	1654.12	1392.18	2539.38
Total amount of energy not-served (kwh)	14	372	18	19	103
Total loss of utility revenue ($)	0.635	18.545	0.84	0.855	5.535
Total outage cost ($)	142	4442	192	190	1390
Total avoided outage cost ($)	9958	8078	9628	9360	8200
Resiliency index	0.9962	0.8997	0.9945	0.9947	0.9718

**Figure 8:**
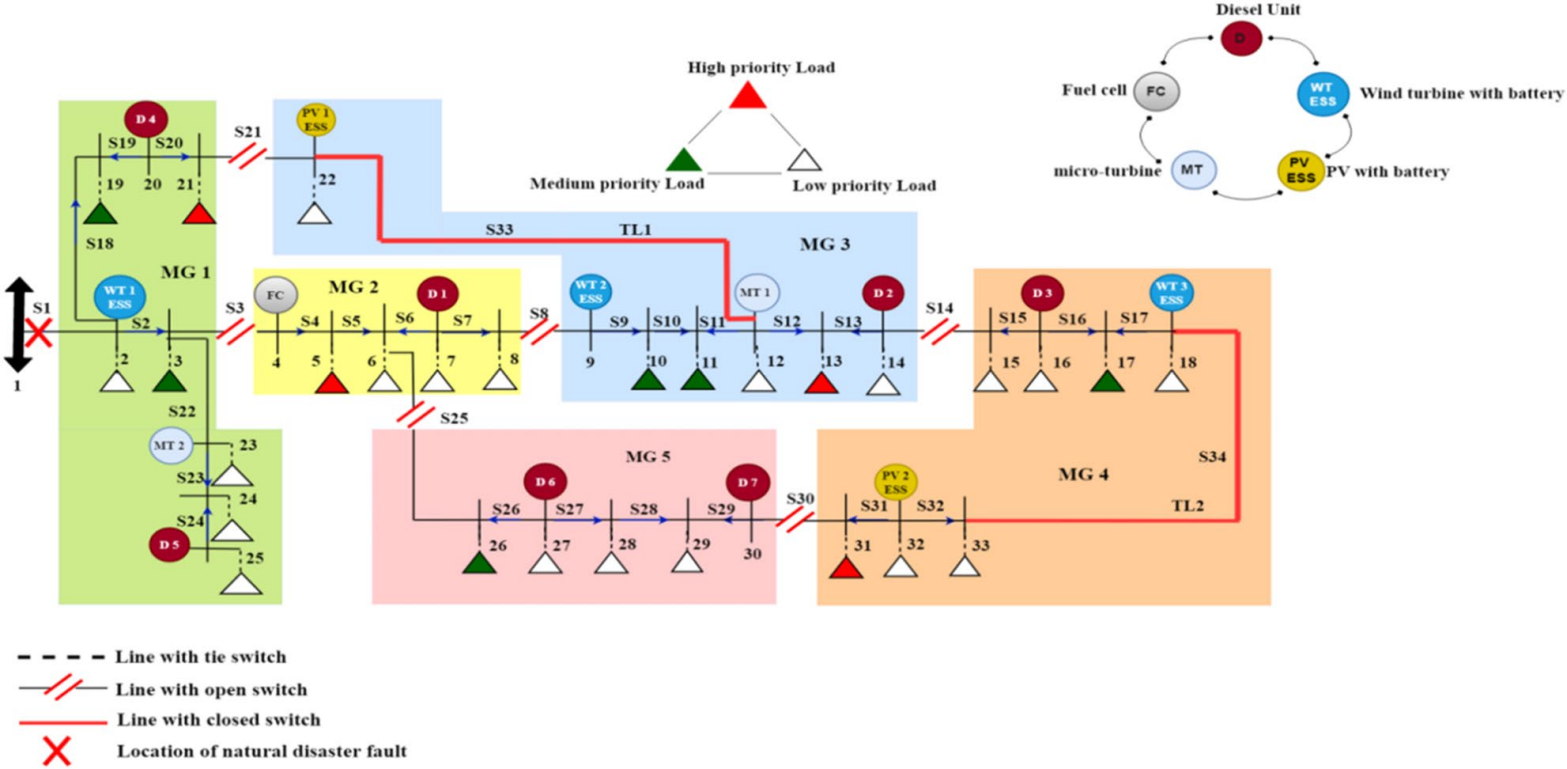
The optimal MG formation scenario with EDRP and Tie-lines.

From Table 2, the scenario with the best objective functions and more resilient out of all scenarios is scenario 1, so it's the best solution for the MG formation for the third case study, as shown in Fig. 8.

A comparison between the results obtained from the proposed method and the results from^12,19,20^ is performed to prove the effectiveness of the proposed method in Table 1. The comparison is subject to the total high-priority restored load and medium-priority loads with/ without DRP program and the resiliency metrics to assess the resilience of the formed MGs and the network under extreme weather incidents.

From Table 5, the proposed method had less energy not-served, loss of utility revenue, outage cost and the most avoided outage cost than the results obtained in^12^. Also, the proposed method could restore the most percentage of high-priority and medium-priority loads with/without DRP than^19^. In addition, the proposed method had the lowest load-shedding and restoration cost, and the resiliency index of the network was the higher than^20^. It can be summarized that the proposed method achieves more resiliency and high performance for microgrid formation strategy in the presence of severe disturbances.

**Table 5:** Comparison between different microgrid formation strategies for enhancing DN Resiliency.

	Objective		High_pr load restoration (%)		Med_pr load restoration (%)		Resiliency metrics				
	LS*(Kwh)	Cost ($)	Without DRP	With DRP	Without DRP	With DRP	Energy not-served (Kwh)	Loss of utility revenue ($)	Outage cost ($)	Avoided outage cost ($)	Resiliency Index
Proposed method	14	1199.52	100	100	79	99	14	0.635	142	9958	0.9962
^12^	–	–	–	–	–	–	3415	355	8537	0	–
^19^	–	–	83	100	16	50	–	–	–	–	–
^20^	4791.66	1812.25	–	–	–	–	–	–	–	–	0.498

LS^*^: Load Shedding, High_pr load restoration: the total amount of high priority loads that are served when applying the proposed method, Med_pr load restoration: the total amount of medium priority loads that are served when applying the proposed method.

Also, a comparison based on the execution time of the proposed method and methods in^11^ and^15^ is performed. The execution time of the proposed method for the modified IEEE 33 bus radial test system are 15, 12, and 10 s for the first, second, and third case studies respectively. In^15^, the execution time of the worst-case scenario is 36 s. However, in^11^, the computational time of the evaluation process for IEEE 33-bus system is 252, 174 s, respectively. So, the solving time of the proposed method is enhanced compared with other related works.

## Conclusion

This paper proposes a novel model for enhancing the cyber-physical DN resiliency-based optimal MG formation method after natural disasters considering EDRP and Tie-line. This method is based on the DGT optimization technique for scenario generation and reduction. Three case studies with and without EDRP and tie-lines are implemented and tested on a modified IEEE 33-bus test system under five scenarios for each case study using Matlab/Script program. The results show the load shedding and the restoration cost are reduced by 97.8% and 75.7%, respectively, by applying the proposed EDRP. In addition, total amount of energy not-served, total loss of utility revenue, and outage penalty cost are decreased by 97.8%, 97.7%, and 77.3%, respectively. Also, the network resiliency index increased by 19%. Furthermore, when applying the EDRP and Tie-line, the load shedding and the restoration cost are minimized by 98.4% and 87.7%, respectively. Moreover, the total amount of energy not-served, total loss of utility revenue, and outage penalty cost are decreased by 98.4%, 98.5%, and 98.5%, respectively. Also, the network resiliency index increased by 19.2%. In future works, modeling the system uncertainties and applying machine learning algorithms to enhance the performance of the proposed method are performed.

## Data Availability

The datasets used and/or analysed during the current study available from the corresponding author on reasonable request.

## Abbreviations

MG: Microgrid
DN: Distribution network
DRERs: Distributed renewable energy resources
ESS: Energy storage system
DG: Diesel generator
PV: Photo-voltaic
WT: Wind turbine
FC: Fuel cell
MT: Micro-turbine
DRP: Demand response program
EDRP: Emergency demand response program
DGT: Darts game theory

## Indices and sets

$n,m,b,r$, q,j: Set of buses, ranging from 1 to ${N}_{bus}$
$g$: Set of distributed renewable energy resources
$dg$: Set of diesel generator units
$e$: Set of energy storage unit
$L$: Set of lines and tie-lines indexed by $l$
$mg$: Set of formable microgrids, ranging from 1 to ${N}_{mg}$
$c$: Index of emergency demand response program step
$d$: Index of emergency demand response program block
$s$: Set of Scenarios
$t$: Index of post-event restoration time
$u$: Set of variables
$v$: Set of events
$\varphi$: Set of neighboring nodes

## Parameters

$${N}^{dg}$$: Number of diesel generator units
$${N}_{mg}$$: Number of formable microgrids
$${N}^{master}$$: Number of master diesel generator units
$${PG}_{g,mg,s,t}^{min}$$ ,$${PG}_{g,mg,s,t}^{max}$$: Minimum and maximum allowed active output (KW)
$${QG}_{g,mg,s,t}^{min}$$ ,$${QG}_{g,mg,s,t}^{max}$$: Minimum and maximum allowed reactive output (kVar)
$${\mathrm{EC}}_{\mathrm{e}}$$: The capacity of ESS
$${\mathrm{SoC}}_{\mathrm{e}}^{\mathrm{min}}$$,$${\mathrm{SoC}}_{\mathrm{e}}^{\mathrm{max}}$$: Minimum and maximum state of charge (kwh)
$${\upmu }^{\mathrm{ch}}, {\upmu }^{\mathrm{dch}}$$: The charging and discharging efficiency of energy storage units
$${C}_{en}$$: The cost of energy ($/kWh)
$${C}_{g,s,t}$$: The operational cost of $${g}^{th}$$ distributed renewable energy resources ($)
$${\tau }_{g}$$: The operational cost coefficient price of distributed renewable energy resources ($/kW)
$${C}_{opf}$$: The outage penalty factors the distribution network operator forces to pay when applying the load-shedding program ($/kW-hr)
$${C}_{EDRP}$$: The multi-step emergency demand response program cost ($)

## Variables

$${P}_{{loss}_{t}}$$: The total power losses (kW) of formable microgrids $$mg$$ at time $$t$$
$${P}_{{shedd}_{t}}$$: The total load shedding (kW) of formable microgrids $$mg$$ at time $$t$$
$${C}_{{rest}_{t}}$$: The total restoration cost ($) of formable microgrids $$mg$$ at time* t*
$${PG}_{g,mg,s,t}$$: Active power generated (kW) of $${g}^{th}$$ distributed renewable energy resources in microgrid $$mg$$
$${QG}_{g,mg,s,t}$$: Reactive power generated (kVar) of $${g}^{th}$$ distributed renewable energy resources in microgrid $$mg$$
$${PG}_{n,mg,s,t}$$: Active power generated (kW) by $$n$$ bus in microgrid $$mg$$
$${QG}_{n,mg,s,t}$$: Reactive power generated (kVar) by $$n$$ bus in microgrid $$mg$$
$${PD}_{n,mg,s,t}$$: Active power demand (kW) at bus $$n$$ in microgrid $$mg$$
$${QD}_{n,mg,s,t}$$: Reactive power demand (kVar) at bus $$n$$ in microgrid $$mg$$
$${P}_{m}^{inf}$$: The inflexible active power (kW) at bus $$m$$
$${Q}_{m}^{inf}$$: The inflexible reactive power (kVar) at bus $$m$$
$${PD}_{b,mg,s,t}^{recovered}$$: The recovered active power demand (kW) at bus $$b$$
$${P}_{e,mg,s,t}^{ch},{P}_{e,mg,s,t}^{dch}$$: The charging and discharging power (kW) of the $${e}^{th}$$ ESS

## Emergency Demand Response Program

${{P}_{EDRP}}_{d,mg,s,t}^{c}$: The value of offered load reduction (kW) of ${d}^{th}$ EDRP blocks
${EDRP}_{d,mg,s,t}^{c}$: Maximum load reduction of ${c}^{th}$ step of EDRP
${EDRP}_{d,mg,s,t}^{min}$: Minimum load reduction of EDRP
${k}^{c}$: The coefficient of the adjusted demand reduction with a step of EDRP
${k}^{min}$: The coefficient of the minimum allowed demand reduction

## Line & bus

${\alpha }_{n,mg}$: Binary variables of buses
${N}_{bus}$: Number of buses
${r}_{n,m} \text{and} {x}_{n,m}$: Resistance and reactance between bus* n* and bus* m*
${I}_{n,m}$: Current between bus $n$ and bus $m$
${\omega }_{b}^{pr}$: The priority of load at bus $b$
${\partial }_{l}$: ${\vartheta }_{l}$: The set of start and end points of line $l$
${Line}_{l}^{initial}$: The initial status of lines
${Bus}_{n}^{initial}$: The initial status of lines
${\beta }_{l,mg}$: ${\rho }_{l,n,r}$: Binary variables of lines
${P}_{n,m}^{flow}$: The active power flow between bus $n$ and $m$
${Q}_{n,m}^{flow}$: The reactive power flow between bus $n$ and $m$
${V}_{n},{V}_{m}$: The voltage at bus $n$ and bus $m$
${\left|V\right|}_{m}^{min}$: ${\left|V\right|}_{m}^{max}$: Minimum and maximum voltage magnitude at bus $m$
${\left|V\right|}_{m}$: The voltage magnitude at bus $m$

## Darts Game Theory

${N}_{p}$: Number of players
${Ft}_{best}$: The value of best fitness function
${Y}_{best}$: The value of best variables
${Ft}_{worst}$: The value of worst fitness function
${Y}_{worst}$: The value of worst variables
${Ft}^{n}$: The fitness function's normalized value
${P}_{i}$: The probability function of each scenario
$S$: The score of the player

## Resiliency Metrics

$$ENS$$: The total amount of energy not-served (kWh)
$${E}_{mg}^{shedd}$$: The total amount of energy not served (kWh) per MG $$mg$$
$${C}_{LOUR,s}$$: The total loss of utility revenue per scenario $$s$$ ($)
$${C}_{op,s}$$: The total outage penalty cost ($)
$${C}_{avd,s}$$: The total avoided outage cost ($)
$${RI}_{s}^{Net}$$: The network resilience index

## References

[1] Espinoza O, Tiwary A (2021). Assessment of autonomous renewable energy system operability under extreme events and disasters. Sustainable Energy Technol. Assessments.

[2] Yang B, Ge S, Liu H, Li J, Zhang S (2022). Resilience assessment methodologies and enhancement strategies of multi-energy cyber-physical systems of the distribution network. IET Energy Syst. Integr..

[3] Hussain A, Bui V, Kim H (2019). Microgrids as a resilience resource and strategies used by microgrids for enhancing resilience. Appl. Energy.

[4] Ti B, Li G, Zhou M, Wang J (2021). Resilience assessment and improvement for cyber-physical power systems under typhoon disasters. IEEE Trans. Smart Grid.

[5] Mahzarnia M, Moghaddam M, Baboli P, Siano P (2020). A review of the measures to enhance power systems resilience. IEEE Syst. J..

[6] Wang Y, Rousis A, Strbac G (2020). On microgrids and resilience: A comprehensive review on modeling and operational strategies. Renew Sustainable Energy Rev.

[7] Shi Q, Liu W, Zeng B, Hui H, Li F (2022). Enhancing distribution system resilience against extreme weather events: Concept review, algorithm summary, and future vision. Electrical Power Energy Syst..

[8] Chen C, Wang J, Qiu F, Zhao D (2015). Resilient distribution system by microgrids formation after natural disasters. IEEE Syst. J..

[9] Zhua J, Yuana Y, Wang W (2020). An exact microgrid formation model for load restoration in resilient distribution system. Int. J. Electr. Power Energy Syst.

[10] Ding T, Lin Y, Bie Z, Chen C (2017). A resilient microgrid formation strategy for load restoration considering master-slave distributed generators and topology reconfiguration. Appl. Energy.

[11] Jalilpoor, K., Taghi, M., Azad, S., Sayadi, Z. Resilient energy management incorporating energy storage system and network reconfiguration: A framework of cyber-physical system. *IET Gen., Trans. Distrib.*, 10.1049/gtd2.12478.

[12] Galvana E, Mandal P, Sang Y (2020). Networked microgrids with roof-top solar PV and battery energy storage to improve distribution grids resilience to natural disasters. Int. J. Electrical Power Energy Syst..

[13] Kizito R, Liu Z, Li X, Sun K (2022). Multi-stage stochastic optimization of islanded utility-microgrids design after natural disasters. Oper. Res. Perspect..

[14] Bahrami M, Vakilian M, Farzin H, Lehtonen M (2021). Multi-step island formation and repair dispatch reinforced by mutual assistance after natural disasters. Int. J. Electr. Power Energy Syst..

[15] Bahrami M, Vakilian M, Farzin H, Lehtonen M (2021). A stochastic framework for optimal island formation during two-phase natural disasters. IEEE Syst. J..

[16] Gilani M, Kazemi A, Ghasemi M (2020). Distribution system resilience enhancement by microgrid formation considering distributed energy Resources. Energy.

[17] Abessi A, Jadid S, Salama M (2020). A new model for a resilient distribution system after natural disasters using microgrid formation and considering ICE cars. IEEE Access.

[18] Ajoulabadi A, Ravadanegh S, Mohammadi-Ivatloo B (2020). Flexible scheduling of reconfigurable microgrid-based distribution networks considering demand response program. Energy.

[19] Kahnamouei AS, Lotfifard S (2021). Enhancing resilience of distribution networks by coordinating microgrids and demand response programs in service restoration. IEEE Syst. J..

[20] Gilani MA, Dashti R, Ghasemi M, Amirioun MH, Shafie-khah M (2022). A microgrid formation-based restoration model for resilient distribution systems using distributed energy resources and demand response programs. Sustainable Cities Soc..

[21] Lynch M, Nolan S, Devine M, O'Malley M (2019). The impacts of demand response participation in capacity markets. Appl. Energy.

[22] Imania M, Niknejad P, Barzegaran MR (2018). The impact of customers' participation level and various incentive values on implementing emergency demand response program in microgrid operation. Int. J. Electr. Power Energy Syst..

[23] Dorahaki S, Dashti R, Shaker H (2020). Optimal outage management model considering emergency demand response programs for a smart distribution system. Appl. Sci..

[24] Dehghani M, Montazeri Z, Givi H, Guerrero J, Dhiman G (2021). Network reconfiguration and distributed energy resource scheduling for improved distribution system resilience. Int. J. Electr. Power Energy Syst..

[25] Dehghani M, Montazeri Z, Givi H, Guerrero JM, Dhiman G (2020). Darts Game Optimizer: A New Optimization Technique Based on Darts Game. International Journal of Intelligent Engineering and Systems.

[26] Najafi J, Peiravi A, Anvari-Moghaddam A, Guerrero J (2020). An efficient interactive framework for improving resilience of power-water distribution systems with multiple privately-owned microgrids. Int. J. Electr. Power Energy Syst..

[27] Ibrahim M, Alkhraibat A (2020). Resiliency assessment of microgrid systems. Appl. Sci..

[28] Ahmadi SE, Rezaei N (2020). A new isolated renewable based multi microgrid optimal energy management system considering uncertainty and demand response. Int. J. Electrical Power Energy Syst..

[29] Iria J, Heleno M, Cardoso G (2019). Optimal sizing and placement of energy storage systems and on-load tap changer transformers in distribution networks. Appl. Energy.

